# Experimentally Induced Stress Validated by EMG Activity

**DOI:** 10.1371/journal.pone.0095215

**Published:** 2014-04-15

**Authors:** Rosan Luijcks, Hermie J. Hermens, Lonneke Bodar, Catherine J. Vossen, Jim van. Os, Richel Lousberg

**Affiliations:** 1 Department of Psychiatry and Psychology, Maastricht University, Maastricht, The Netherlands; 2 Roessingh Research and Development, Enschede, The Netherlands; 3 Department of Anesthesiology and Pain Medicine, Maastricht University Medical Centre, Maastricht, The Netherlands; 4 King's Health Partners, King's College London, Department of Psychosis Studies, Institute of Psychiatry, London, United Kingdom; University of Rome Foro Italico, Italy

## Abstract

Experience of stress may lead to increased electromyography (EMG) activity in specific muscles compared to a non-stressful situation. The main aim of this study was to develop and validate a stress-EMG paradigm in which a single uncontrollable and unpredictable nociceptive stimulus was presented. EMG activity of the trapezius muscles was the response of interest. In addition to linear time effects, non-linear EMG time courses were also examined. Taking into account the hierarchical structure of the dataset, a multilevel random regression model was applied. The stress paradigm, executed in N = 70 subjects, consisted of a 3-minute baseline measurement, a 3-minute pre-stimulus stress period and a 2-minute post-stimulus phase. Subjects were unaware of the precise moment of stimulus delivery and its intensity level. EMG activity during the entire experiment was conform *a priori* expectations: the pre-stimulus phase showed a significantly higher mean EMG activity level compared to the other two phases, and an immediate EMG response to the stimulus was demonstrated. In addition, the analyses revealed significant non-linear EMG time courses in all three phases. Linear and quadratic EMG time courses were significantly modified by subjective anticipatory stress level, measured just before the start of the stress task. Linking subjective anticipatory stress to EMG stress reactivity revealed that subjects with a high anticipatory stress level responded with more EMG activity during the pre-stimulus stress phase, whereas subjects with a low stress level showed an inverse effect. Results suggest that the stress paradigm presented here is a valid test to quantify individual differences in stress susceptibility. Further studies with this paradigm are required to demonstrate its potential use in mechanistic clinical studies.

## Introduction

Chronic stress is a risk factor for both somatic outcomes, such as hypertension [1], [2], [3], coronary heart disease [4], peptic ulcers [5], [6], [7] and asthma [8], and mental health outcomes including chronic ‘benign’ pain [9], [10], anxiety [11], [12], [13], PTSD [7], [14], depression [13], [15] and burn-out [15], [16]. In addition, work-related exposures such as repetitive work or mental stress during work, increase the risk for stress-related pain [17], [18], [19], [20], [21], [22], [23].

After Selye's theoretical and experimental pioneering work, more elaborate definitions of stress were proposed, including the concept of Levine and Ursin that makes a distinction between three elements: the input (stress stimuli), the processing systems (including the subjective experience of stress) and the output (stress responses) [24]. Stress, like pain, is best conceptualized as a theoretical construct, which is not directly measurable. A biopsychosocial approach to stress measurement may be useful, focusing on biological (somatic), psychological (behavioral) and social (cultural) dimensions of stress. To cover the three dimensions of stress, measurement should include both subjective measures (self-report questionnaires) and (psycho)physiological measures. The experience of stress is generally accompanied by an increased level of arousal and may lead to a number of physiological reactions, such as acceleration of the heart rate, pupil dilatation, increased galvanic skin response and increased finger pulse volume. Muscle activity measured with electromyography (EMG) also is sensitive to stress. The majority of experimental, clinical and field studies report an increase in EMG activity [25], [26], [27], [28], [29], [30], [31], [32], [33], [34]. Increased EMG activity is prominent in the trapezius muscles, particularly if the stressor is personally relevant. Interestingly, however, some studies do not report such a relationship [35], [36], [37].

A well-known caveat in stress experiments is that the procedure of stress-induction is not under adequate experimental control. There are many possibilities to induce stress experimentally [38]. Examples include solving a mental arithmetic task, the Stroop word color test, examination stress, public speaking tests (e.g. the Trier Social Stress Test) or a combination of different stress tasks [39], but also paradigms in which subjects have to undergo an unpleasant physical stimulus, such as the cold pressure test [40] or electric shock test [35], [41], [42], [43]. It is unlikely that the above stress paradigms are unpredictable and/or uncontrollable to the same degree, which is relevant, given that one of the most crucial stress modifying factors is the degree to which a stimulus can be predicted and/or controlled [44], [45], [46], [47], [48]. In the stress paradigm of repeated (nociceptive) stimuli, the measurement of the direct stress response of each stimulus may be confounded by the phenomenon of habituation. In addition, experimental stress tasks may be confounded if accompanied by painful procedures not associated with the experiment itself, such as venipuncture. As a final critical point, it is important to note that there is an essential difference between a cognitive and a physical stressor. Most stress paradigms provide either a cognitive or a physical stressor. Studies providing both type of stressors at the same time are scarce (e.g. [25]).

Led by these considerations, the goal of this study was to develop a novel and robust experimental stress paradigm according to the following principles. First, the stress paradigm should present a distinct cognitive and a distinct physical stressor. Second, it should include elements of uncontrollability and unpredictability, and third, it should be simple to administer, independent of illness status, educational level, motivation and other factors. To this end, a dual stress induction was introduced, consisting of (i) the announcement of imminent receipt of a single uncontrollable and unpredictable electric shock, thus inducing a cognitive anticipatory stress phase, followed by (ii) a nociceptive stimulus.

Within this experiment, three phases can be distinguished: (i) an anticipation, pre-stimulus phase, during which subjects are anticipating an electric shock in a relatively uncontrolled/unpredictable situation; (ii) an immediate post-stimulus phase, showing the initial response to the nociceptive stimulus and (iii) a return to baseline phase, which can be described as the time required to achieve a comparable level of baseline physiological activity. Based on this paradigm, stress reactivity can be quantified and stored, reflecting individual differences in stress susceptibility.

The clear advantage of this paradigm is that the presentation of the physical stimulus has two functions: i) it makes the examination of a physical stressor possible and ii) it allows to demarcate the period of mental stress induction, since subjects were instructed that there would only be one physical stimulus.

A second goal of the study was to investigate in detail the muscle activation response during the stress condition. Experimental muscle response stress paradigms typically examine the linear time-related post-stimulus effects. This is logical, given the expectation that EMG activity increases while tension is building up in the anticipatory pre-stimulus phase. A linear time course suggests that the highest EMG activity would be reached just before receiving the stimulus, but considering the fact that the subjects receive an unpredictable stimulus, a more complex combination of linear and non-linear time effects may be more appropriate, for example in the form of a higher order polynomial function, which reaches a maximum. Modeling non-linear time effects is also relevant for the post-stimulus phase. Modeling an inverse time function (1/time) is desirable, as this function portrays an initial sharp decrease or increase, followed by a plateau, which is applicable to the post-stimulus phase. EMG activity is typically measured by a computed average over a complete experimental condition. Thus, condition effects are tested by a comparison of the condition means. To this end, linear regression or ANOVA analyses are used. However, measuring EMG over time implicates a hierarchical structure of the data, in which consecutive time elements are nested within subjects. This hierarchical structure needs to be taken into account using multilevel random regression procedures [49].

We hypothesized that in comparison to baseline, EMG activity would increase during the pre-stimulus anticipation period, and would show a return to baseline in the post-stimulus period. In addition to the main hypothesis, we expected (i) non-linear EMG effects during pre- and post-stimulus stress periods, hypothesizing presence of both quadratic and inverse effects; (ii) asymmetric (left-right) EMG activity in the trapezius muscles, since we presented the electric stimulus unilaterally, on the left side; and (iii) given the multidimensional nature of stress, a positive association between subjective anticipatory stress on the one hand and EMG activity on the other. Further, in addition to a main effect, an interaction between the two different phases of the stress task and subjective anticipatory stress was expected. Since subjective stress is assumed to correlate positively with EMG activity, a larger increase in EMG activity within the pre-stimulus phase was expected in subjects with high levels of anticipatory stress, as compared to subjects with low levels of anticipatory stress.

## Materials and method

### Ethics Statement

The study was conducted according to the principles of the Declaration of Helsinki and was approved by the medical ethics committee of the Academic Hospital Maastricht and Maastricht University (METC azM/UM, Maastricht). Before the start of the experiment, subjects provided written informed consent.

### Subjects

Seventy right-handed subjects (44 females and 26 males) participated in the study. Their age ranged from 18 to 65 years. Exclusion criteria were structural use of antipsychotics, anti-epileptics or anxiolytics during the past year or structural use of alcohol (>10 u/day). Subjects were asked to refrain from alcohol-containing consumptions the evening before and to refrain from caffeine-containing consumptions three hours prior to the experiment.

### Electroshocker and stimuli

An electro-shocker (type Shocko-100-AA-20, developed by Maastricht Instruments BV and approved for usage in experimental studies) was used to deliver electroshocks (see also [50]). Stimuli were electrical pulses of 10 milliseconds duration, administered intracutaneously on the top of the middle finger of the non-dominant left hand, as described by Bromm and Meier [51]. The sensation and pain threshold were determined by gradually increasing the intensity of the stimulus, starting at zero intensity. The first intensity that was consciously experienced was defined as the sensation threshold, the first intensity experienced as painful was defined as the pain threshold. This procedure was repeated three times in order to obtain a reliable estimate. The intensity of the electric stimulus applied during the experiment was computed for each subject individually. The intensity of the actually delivered stress stimulus during the experiment was calculated as follows:

Actually delivered stress stimulus  =  pain threshold + 0.25*(pain threshold − sensation threshold)

As shown in a previous experiment, this intensity level was experienced as painful by all subjects, albeit still acceptable [50].

### Procedure

EMG-, and ECG-electrodes as well as the shock electrode were attached. EMG-electrodes were attached on the left and right trapezius muscle.

The baseline measurement was 3 minutes. After determination of the individual pain threshold, subjects were instructed that they would receive a single electric shock sometime during a 5-minute period. The experimenter pointed out that the precise moment of stimulus delivery and its intensity level would be determined by a personal computer. In addition, subjects were told that stimulus intensity might vary between the sensation threshold and a level clearly above the pain threshold. Just before the start of the task, subjects had to answer the following question on a 10 point Likert scale: How much stress do you experience at this moment, awaiting the stimulus? Subjects were instructed to keep both hands on the table, palms down, and not to close their eyes during the whole measurement period. In fact, all subjects received the experimental stimulus at exactly t = 3 minutes. The whole procedure was controlled by the software program “Presentation 0.71” (Neurobehavioral Systems).

### Psychophysiological recordings

All recordings were conducted in an electrically and sound-shielded cubicle (7.1 m^2^). EMG activity was recorded from the left and right upper trapezius muscle. Electrodes were centered on a point 2 cm lateral to the midpoint between the acromion process and spinous process of the seventh cervical vertebra, using Ag/AgCl electrodes. A reference electrode was placed over the spinous process of the seventh cervical vertebra. Cardiac activity was recorded with a standard 3 lead ECG. All electrodes were fixed using 10–20 conductive paste. Brainvision BrainAmp Research Amplifier was used for all recordings. ECG and EMG were sampled with 1000 Hz.

### Offline dataprocessing

EMG data was filtered offline (low pass 0.5 Hz, high pass 250 Hz, 50 Hz notch filter) and segmented into epochs of 500 ms. Raw data were visually inspected for artifacts and, if found, excluded from further analyses. EMG activity was corrected for ECG activity: the variance due to ECG activity was removed from the uncorrected EMG variable, using regression analysis. Next, for each 500 ms epoch, the root mean square value was calculated followed by a logarithmic transformation to preserve a normal distribution.

### Statistical analysis

Given the hierarchical structure of the EMG dataset, consisting of epochs (level 1) that are clustered within individuals (level 2), multilevel random regression analyses were performed. EMG activity served as the dependent variable. Epoch number and condition, coded in two dummy variables contrasting baseline and post-stimulus versus pre-stimulus, served as independent variables in the basic model. Number of segment was included in order to investigate the linear effect over time. In addition to this linear time effect, a quadratic (epoch*epoch) and inverse effect (1/epoch) were added.

In order to test which covariance structure yielded the best fit for our dataset, various covariance structures were tested. Scaled Identity turned out to be significantly better than that of its competitors, namely compound symmetry (CS) and AR1. All models were tested with a random intercept. All statistical analyses were performed using SPSS 20.0. P-values below 0.05 were considered to be statistically significant.

## Results

Due to protocol violations (eyes closed, movements), 6 subjects were excluded from the analyses, leaving n = 64 analyzable participants (40 females, 24 males).

Before analyzing the psychophysiological reactivity of the stress task, the amount of anticipatory stress experienced was analyzed. The mean score on the subjective stress item was 3.6 (SD  =  2.61) with a score range from 0 to 10. As expected, the mean score was significantly different from 0 (t = 10.68, p<0.0001), indicating that, on average, subjects experienced mild to moderate stress. Individual scores ranged from 0 to 10, indicative of large between-subject differences in experience of anticipatory stress.

### EMG activity during the three experimental phases

Table 1 shows the results comparing the average left and right trapezius EMG activity for the baseline, pre-stimulus and post-stimulus periods. Mean EMG activity during the pre-stimulus period was significantly higher compared to baseline period (left trapezius muscle t  =  32.13, p<0.001; right trapezius muscle t =  31.33, p<0.001), whereas mean EMG activity during the post-stimulus period was significantly lower compared to pre-stimulus period (left t =  −6.70, p<0.001; right t =  −9.62, p<0.001). An asymmetric (left-right) reaction in EMG activity was observed. The overall tension of the left trapezius muscle was higher than the tension of the right trapezius muscle during the whole experiment (p<10^−6^)(Table 1). The random intercepts of the models were significant (all p's <0.001), indicating that EMG activity level at the start of each phase varied significantly between subjects.

**Table 1:** Mean EMG activity (in RMS) for baseline period, pre-stimulus period and post-stimulus period.

Period	Left trapezius EMG activity (mean and SD in log RMS)	Right trapezius EMG activity (mean and SD in log RMS)
Baseline	µ = 1.29 [σ = 0.29]	µ = 1.24 [σ = 0.30]
Pre-stimulus	µ = 1.38 [σ = 0.30]	µ = 1.34 [σ = 0.31]
Post-stimulus	µ = 1.36 [σ = 0.29]	µ = 1.30 [σ = 0.32]

The linear time*condition interaction effect of EMG activity was investigated. As hypothesized, EMG activity showed a significant increase during the pre-stimulus period compared to the baseline period, for both the left (t = 2.31, p = 0.021) and the right (t = 2.46, p = 0.014) trapezius muscle. In comparison with the pre-stimulus period, a very significant contrasting (decreasing EMG activity) linear time*condition interaction was observed in the post-stimulus phase, again both left (t =  −15.84, p = <0.0001) and right (t =  −12.38, p = <0.0001).

**Figure 1:**
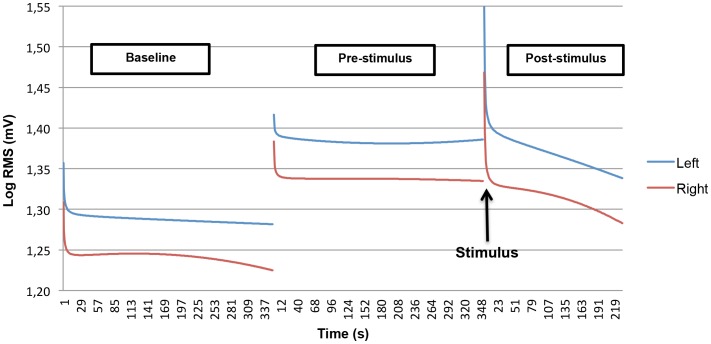
Modeled non-linear EMG activity in trapezius muscle for baseline, pre- and post-stimulus period.

Non-linear EMG time effects were modeled *post-hoc*, by including a quadratic and inverse component as predictor variables in the multilevel model. The results of this model are shown in figure 1.

An inverse effect was observed at all periods, consisting of a rapid initial decline of EMG values immediately after the start of each period, followed by a plateau phase. The inverse and parabolic effects were significantly different between the pre- and post-stimulus period, for both the left (t = 5.53, p<0.0001) and right (t = 4.20, p<0.0001) trapezius muscle. With regard to the parabolic component, there was a statistically significant pre-post difference, only for the right trapezius muscle (t = −2.46, p = 0.014). This quadratic difference indicates that the parabolic decrease in the EMG activity of the right trapezius muscle in the post-stimulus period was larger than the parabolic decrease in the pre-stimulus period.

Figure 1 shows that a complete return of EMG activity to baseline level (i.e. mean EMG activity in the baseline period) did not take place in the post-stimulus phase.

Finally, a series of post-hoc analyses were carried in order to investigate possible main sex differences in EMG activity. No significant differences could be demonstrated.

### Response to the nociceptive stimulus

**Figure 2:**
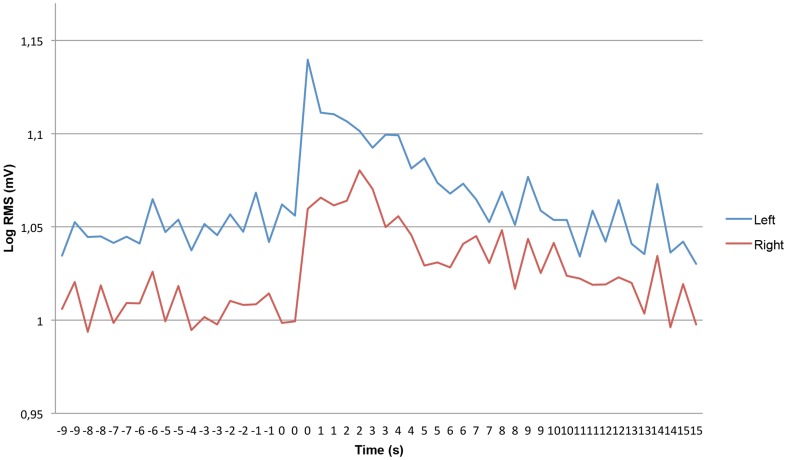
Direct stimulus response, showing a time interval of 10 seconds pre-stimulus to 15 seconds post-stimulus.

Figure 2 shows the short-term effect of the experimental stimulus on EMG activity for a time window ranging from 10 seconds pre-stimulus to 15 seconds post-stimulus. The immediate EMG effect of the nociceptive stimulus consisted of a sharp increase, both left and right, followed by a pronounced decrease. The time required to reach an EMG activity level equal to or lower than the level just before the stimulus is about 15 seconds (Fig. 2).

### Subjective anticipatory stress and EMG activity

Including the non-linear time components (quadratic and inverse) in the model, it was tested whether the mean EMG activity level in pre- and post-stimulus phase was independently associated with the subjective anticipatory stress level. Contrary to the hypothesis, this appeared to be the case in neither the pre-stimulus nor the post-stimulus period, for both the left and the right trapezius muscle (all p's>0.70).

**Figure 3:**
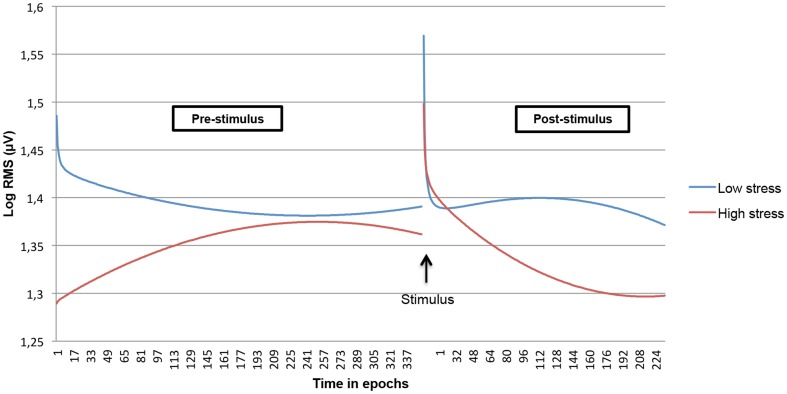
Modeled non-linear time course during the stress task interacted with subjective anticipatory stress for the left trapezius muscle. Low and high stress levels are contrasted.

**Figure 4:**
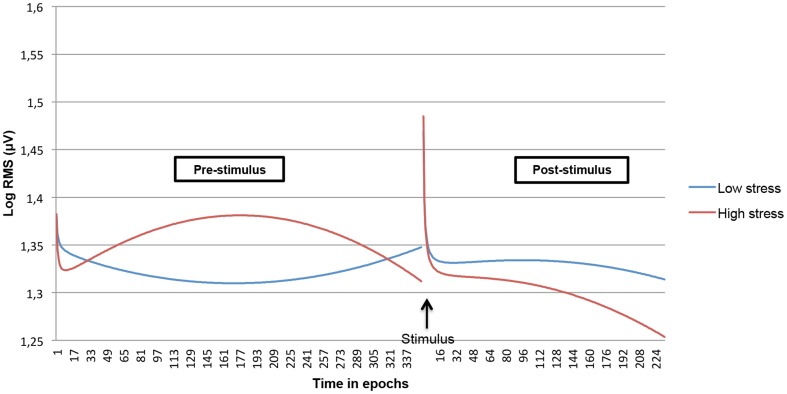
Modeled non-linear time course during the stress task interacted with subjective anticipatory stress for the right trapezius muscle. Low and high stress levels are contrasted.

We examined whether both linear and non-linear time courses within the pre- and post-stimulus period interacted with subjective anticipatory stress. In order to visualize the interaction effects as contrasting as possible, only the predicted EMG time course of the highest anticipatory stress score (10) and the lowest anticipatory stress score (0) was plotted. This revealed strong differences in the (non-linear) time course of the stress task between the extremes (Fig.3 and 4). Testing the hypothesized greater level of EMG activity in subjects experiencing more stress in the pre-stimulus phase revealed that both left and right trapezius muscle showed this effect: subjects with the highest stress level (score 10), showed a quadratic initial increase of EMG activity during the pre-stimulus period, while subjects with a zero stress level tended to display an initial decrease during the pre-stimulus period. The multilevel models, including the anticipatory stress scale as a continuous variable, showed highly significant linear and parabolic effects (all p's<0.0001). No interaction effects for the inverse time component were found.

The results of a final *post hoc* analysis, concerning the post-stimulus period, were less conclusive. Whereas in the left trapezius muscle significant linear and parabolic interaction effects were apparent, these effects were not evident in the right trapezius muscle.

## Discussion and conclusion

### General EMG effects

The key finding of this study was that, in agreement with the *a priori* hypotheses, significant differences in mean EMG activity were observed (in both left and right trapezius), between the three experimental phases of a novel experimental stress paradigm including a cognitive and a physical stressor. In addition, a clear response to the experimental nociceptive stimulus was demonstrated. Mean EMG activity level was higher in the pre-stimulus period compared to both the baseline and the post-stimulus phase. Further, during all three periods, a linear time course in EMG was present. During the pre-stimulus phase there was a linear increase in EMG activity compared to baseline, followed by a decreasing linear course during the post-stimulus phase.

Non-linear EMG time courses were observed. The pronounced inverse effect, characterized by a rapid decline just after the start of each of the three phases, may be explained as follows: with respect to the baseline period and the pre-stimulus phase, the EMG recording started immediately after instructions were given by the experimenter. This ‘event’ may have caused an increase in EMG activity, which rapidly disappeared after the start of the measurement. The inverse effect at the start of the post-stimulus phase is attributed to the stimulus reactivity. In addition to these inverse effects, parabolic effects were also modeled. In contrast to the inverse effects, no parabolic time component in EMG activity was significant.

In all models, a significant random intercept was observed, indicating that there was random (intercept) variability in EMG starting level between subjects. This so-called intercept variability in ‘habitual EMG level’ is a well-known phenomenon in (clinical) practice. It can be concluded that multilevel analyses are required when investigating EMG activity in this type of stress experiment.

Anticipatory stress level, measured just before the start of the stress task, was mild to moderate on average. Given subjective experience of stress, with parallel evidence of changes in EMG activity, suggests that the stress task was validated. In addition, EMG activity during the three phases was demonstrated in the expected direction given the dual nature (the cognitive and nociceptive stress elements) of the stress task. The relative increase (compared to the baseline period) during the pre-stimulus phase can be regarded as the cognitive stress response. The immediate, sharp increase after the experimental stimulus can be regarded as the nociceptive stress response.

### Influence of subjective stress on EMG activity

There was no association between the level of subjective anticipatory stress and the *mean* EMG activity level, not even during the pre-stimulus phase. Apparently, the intuitive assumption that subjective stress is expressed in a general rise in EMG activity does not apply. Interpersonal differences in this relationship, related to third variables including personality factors, may create heterogeneity. Some examples of these potentially relevant personality characteristics are maladaptive coping strategies such as catastrophizing [52], state anxiety level [53] and specific personality traits, such as neuroticism [13]. In this respect, it becomes clear that in a proper analysis of EMG variability, a multidimensional perspective is necessary.

When modeling the quadratic and inverse time effects, it was found that the EMG non-linear time course during the pre-stimulus phase was moderated by anticipatory stress level. For both left and right trapezius muscle in the pre-stimulus phase, EMG activity developed exactly in the direction which was predicted: subjects with high anticipatory stress showed an initial increase, whereas subjects with low anticipatory-stress showed an opposite EMG time course. The observation that the top of the fitted parabola was reached within the pre-stimulus phase, may be associated with the unpredictability, i.e. the uncertainty about the exact point of time the stimulus would be received.

Although different EMG time courses were observed during the post-stimulus phase, there was no consistency between the left and right trapezius muscle. This left-right inconsistency in the post-stimulus phase may be explained by the fact that the stimulus was applied to only one (left) hand, leading to an asymmetry in EMG activity. More experimental research to unravel this issue is required.

Some methodological issues are apparent. First, since return to baseline has been suggested as a useful stress parameter [10], [54], it would have been interesting to carry out analyses on this measure. The results made clear (figure 1), however, that the post-stimulus period was too short to reach a complete return to the EMG activity level of the baseline phase. Therefore, in a future experiment, the length of the post-stimulus period should be prolonged. A second point is that one could argue that the response to the physical stimulus is a superimposed reaction on an existing mental stress state, and thus might be biased. This is true, but the ideal situation, in which an experimentally controlled physical stressor is presented without the knowledge of the subject, might be considered as unethical. Further, stress experiments using a series of physical stimuli, implicitly contain a mental stress element. In other words, it seems impossible to set up a physical-stress paradigm without also inducing a mental stressor. Another critical note pertains the generalizability of the results of the stress task: the current paradigm may be considered as a rigid stress task, performed in a laboratory setting. This undoubtedly has its limitations to the generalization to daily life stress situations. On the other hand, the exact timing of the physical stressor and the identical environment for all subjects facilitates the examination of individual variability of stress reactivity and aides in linking stress mechanisms to subjective experienced stress levels.

In sum, a new stress paradigm has been developed which contains a dual stress induction, presenting both a mental stressor as well as a physical stressor. Additionally, the stress task is straight-forward and easy to administer in a broad population, independent of illness status, educational level, motivation and other factors. The results of the analyses provide evidence for the validity of the stress task. The present paradigm may be used in future studies on fundamental stress mechanisms. Dependent on the research question, other stress-related (psycho)physiological parameters can be incorporated. This stress task may also be used to assess the relationship between experimentally induced psychophysiological stress reactivity and a subsequent health outcomes. As such, it may possibly be useful as a diagnostic tool in clinical practice, and contribute to high-risk preventive paradigms.

## References

[1] Jones A, McMillan MR, Jones RW, Kowalik GT, Steeden JA, et al (2012) Adiposity is associated with blunted cardiovascular, neuroendocrine and cognitive responses to acute mental stress. PloS one 7: e39143. 22745709 10.1371/journal.pone.0039143 PMC3380036

[2] Esler M, Eikelis N, Schlaich M, Lambert G, Alvarenga M, et al (2008) Chronic mental stress is a cause of essential hypertension: presence of biological markers of stress. Clinical and experimental pharmacology & physiology 35: 498–502. 18307749 10.1111/j.1440-1681.2008.04904.x

[3] Sparrenberger F, Cichelero FT, Ascoli AM, Fonseca FP, Weiss G, et al (2008) Does psychosocial stress cause hypertension[quest] A systematic review of observational studies. J Hum Hypertens 23: 12–19. 18615099 10.1038/jhh.2008.74

[4] Richardson S, Shaffer JA, Falzon L, Krupka D, Davidson KW, et al (2012) Meta-Analysis of Perceived Stress and Its Association With Incident Coronary Heart Disease. American Journal of Cardiology 110: 1711–1716. 22975465 10.1016/j.amjcard.2012.08.004 PMC3511594

[5] Levenstein S, Kaplan GA, Smith M (1995) Sociodemographic characteristics, life stressors, and peptic ulcer. A prospective study. Journal of clinical gastroenterology 21: 185–192. 8648050 10.1097/00004836-199510000-00004

[6] Levenstein S, Kaplan GA, Smith MW (1997) Psychological predictors of peptic ulcer incidence in the Alameda County Study. Journal of clinical gastroenterology 24: 140–146. 9179731 10.1097/00004836-199704000-00004

[7] Handwerger K (2009) Differential patterns of HPA activity and reactivity in adult posttraumatic stress disorder and major depressive disorder. Harvard review of psychiatry 17: 184–205. 19499418 10.1080/10673220902996775

[8] Wright RJ, Rodriguez M, Cohen S (1998) Review of psychosocial stress and asthma: an integrated biopsychosocial approach. Thorax 53: 1066–1074. 10195081 10.1136/thx.53.12.1066 PMC1745142

[9] Flor H, Birbaumer N, Schugens MM, Lutzenberger W (1992) Symptom-specific psychophysiological responses in chronic pain patients. Psychophysiology 29: 452–460. 1410176 10.1111/j.1469-8986.1992.tb01718.x

[10] Flor H, Turk DC (1989) Psychophysiology of chronic pain: do chronic pain patients exhibit symptom-specific psychophysiological responses? Psychological bulletin 105: 215–259. 2648442 10.1037/0033-2909.105.2.215

[11] Strohle A, Holsboer F (2003) Stress responsive neurohormones in depression and anxiety. Pharmacopsychiatry 36 Suppl 3S207–214. 14677081 10.1055/s-2003-45132

[12] Abelson JL, Curtis GC (1996) Hypothalamic-pituitary-adrenal axis activity in panic disorder. 24-hour secretion of corticotropin and cortisol. Archives of general psychiatry 53: 323–331. 8634010 10.1001/archpsyc.1996.01830040059010

[13] Uliaszek AA, Zinbarg RE, Mineka S, Craske MG, Sutton JM, et al (2009) The role of neuroticism and extraversion in the stress–anxiety and stress–depression relationships. Anxiety, Stress & Coping 23: 363–381. 10.1080/10615800903377264 PMC3690955 19890753

[14] de Kloet CS, Vermetten E, Heijnen CJ, Geuze E, Lentjes EG, et al (2007) Enhanced cortisol suppression in response to dexamethasone administration in traumatized veterans with and without posttraumatic stress disorder. Psychoneuroendocrinology 32: 215–226. 17296270 10.1016/j.psyneuen.2006.12.009

[15] Chrousos GP, Gold PW (1992) The concepts of stress and stress system disorders. Overview of physical and behavioral homeostasis. JAMA: the journal of the American Medical Association 267: 1244–1252. 1538563

[16] Tyssen R, Vaglum P, Gronvold NT, Ekeberg O (2000) The impact of job stress and working conditions on mental health problems among junior house officers. A nationwide Norwegian prospective cohort study. Medical education 34: 374–384. 10760123 10.1046/j.1365-2923.2000.00540.x

[17] Larsman P, Thorn S, Sogaard K, Sandsjo L, Sjogaard G, et al (2009) Work related perceived stress and muscle activity during standardized computer work among female computer users. Work 32: 189–199. 19289872 10.3233/WOR-2009-0805

[18] Lundberg U (1999) Stress responses in low-status jobs and their relationship to health risks: musculoskeletal disorders. Annals of the New York Academy of Sciences 896: 162–172. 10681896 10.1111/j.1749-6632.1999.tb08113.x

[19] Thorn S, Sogaard K, Kallenberg LA, Sandsjo L, Sjogaard G, et al (2007) Trapezius muscle rest time during standardised computer work-a comparison of female computer users with and without self-reported neck/shoulder complaints. Journal of electromyography and kinesiology: official journal of the International Society of Electrophysiological Kinesiology 17: 420–427. 16829137 10.1016/j.jelekin.2006.04.010

[20] Voerman GE, Vollenbroek-Hutten MM, Hermens HJ (2007) Upper trapezius muscle activation patterns in neck-shoulder pain patients and healthy controls. European journal of applied physiology 102: 1–9. 16845552 10.1007/s00421-006-0215-8

[21] Bansevicius D, Westgaard RH, Stiles T (2001) EMG activity and pain development in fibromyalgia patients exposed to mental stress of long duration. Scandinavian journal of rheumatology 30: 92–98. 11324796 10.1080/03009740151095367

[22] Lundberg U, Dohns IE, Melin B, Sandsjo L, Palmerud G, et al (1999) Psychophysiological stress responses, muscle tension, and neck and shoulder pain among supermarket cashiers. Journal of occupational health psychology 4: 245–255. 10431284 10.1037//1076-8998.4.3.245

[23] Rissen D, Melin B, Sandsjo L, Dohns I, Lundberg U (2000) Surface EMG and psychophysiological stress reactions in women during repetitive work. European journal of applied physiology 83: 215–222. 11104063 10.1007/s004210000281

[24] Levine S, Ursin H (1991) What is stress? In: Brown MR, Rivier, C, Koob, G (Eds.), editor. Stress, Neurobiology and Neuroendocrinology. New York: Marcel Decker. pp. 3–21.

[25] Krantz G, Forsman M, Lundberg U (2004) Consistency in physiological stress responses and electromyographic activity during induced stress exposure in women and men. Integrative physiological and behavioral science: the official journal of the Pavlovian Society 39: 105–118. 15759598 10.1007/BF02734276

[26] Tulen JH, Moleman P, van Steenis HG, Boomsma F (1989) Characterization of stress reactions to the Stroop Color Word Test. Pharmacology, biochemistry, and behavior 32: 9–15. 10.1016/0091-3057(89)90204-9 2734355

[27] Lundberg U, Kadefors R, Melin B, Palmerud G, Hassmen P, et al (1994) Psychophysiological stress and EMG activity of the trapezius muscle. International journal of behavioral medicine 1: 354–370. 16250795 10.1207/s15327558ijbm0104_5

[28] Flodgren GM, Crenshaw AG, Gref M, Fahlstrom M (2009) Changes in interstitial noradrenaline, trapezius muscle activity and oxygen saturation during low-load work and recovery. European journal of applied physiology 107: 31–42. 19504120 10.1007/s00421-009-1095-5

[29] Wahlstrom J, Hagberg M, Johnson PW, Svensson J, Rempel D (2002) Influence of time pressure and verbal provocation on physiological and psychological reactions during work with a computer mouse. European journal of applied physiology 87: 257–263. 12111287 10.1007/s00421-002-0611-7

[30] Schleifer LM, Spalding TW, Kerick SE, Cram JR, Ley R, et al (2008) Mental stress and trapezius muscle activation under psychomotor challenge: a focus on EMG gaps during computer work. Psychophysiology 45: 356–365. 18282206 10.1111/j.1469-8986.2008.00645.x

[31] Laursen B, Jensen BR, Garde AH, Jorgensen AH (2002) Effect of mental and physical demands on muscular activity during the use of a computer mouse and a keyboard. Scandinavian journal of work, environment & health 28: 215–221. 10.5271/sjweh.668 12199422

[32] Wahlstrom J, Lindegard A, Ahlborg G Jr, Ekman A, Hagberg M (2003) Perceived muscular tension, emotional stress, psychological demands and physical load during VDU work. International archives of occupational and environmental health 76: 584–590. 12898271 10.1007/s00420-003-0454-5

[33] Finsen L, Sogaard K, Jensen C, Borg V, Christensen H (2001) Muscle activity and cardiovascular response during computer-mouse work with and without memory demands. Ergonomics 44: 1312–1329. 11900421 10.1080/00140130110099065

[34] Svebak S, Anjia R, Karstad SI (1993) Task-induced electromyographic activation in fibromyalgia subjects and controls. Scandinavian journal of rheumatology 22: 124–130. 8316773 10.3109/03009749309099256

[35] Noteboom JT, Barnholt KR, Enoka RM (2001) Activation of the arousal response and impairment of performance increase with anxiety and stressor intensity. Journal of applied physiology 91: 2093–2101. 11641349 10.1152/jappl.2001.91.5.2093

[36] Blangsted AK, Sogaard K, Christensen H, Sjogaard G (2004) The effect of physical and psychosocial loads on the trapezius muscle activity during computer keying tasks and rest periods. European journal of applied physiology 91: 253–258. 14569401 10.1007/s00421-003-0979-z

[37] Bansevicius D, Westgaard RH, Jensen C (1997) Mental stress of long duration: EMG activity, perceived tension, fatigue, and pain development in pain-free subjects. Headache 37: 499–510. 9329233 10.1046/j.1526-4610.1997.3708499.x

[38] Biondi M, Picardi A (1999) Psychological stress and neuroendocrine function in humans: the last two decades of research. Psychotherapy and psychosomatics 68: 114–150. 10224513 10.1159/000012323

[39] Reinhardt T, Schmahl C, Wüst S, Bohus M (2012) Salivary cortisol, heart rate, electrodermal activity and subjective stress responses to the Mannheim Multicomponent Stress Test (MMST). Psychiatry research 198: 106–111. 22397919 10.1016/j.psychres.2011.12.009

[40] Schwabe L, Haddad L, Schachinger H (2008) HPA axis activation by a socially evaluated cold-pressor test. Psychoneuroendocrinology 33: 890–895. 18403130 10.1016/j.psyneuen.2008.03.001

[41] Breznitz S, Ben-Zur H, Berzon Y, Weiss DW, Levitan G, et al (1998) Experimental induction and termination of acute psychological stress in human volunteers: effects on immunological, neuroendocrine, cardiovascular, and psychological parameters. Brain, behavior, and immunity 12: 34–52. 10.1006/brbi.1997.0511 9570860

[42] Weisse CS, Pato CN, McAllister CG, Littman R, Breier A, et al (1990) Differential effects of controllable and uncontrollable acute stress on lymphocyte proliferation and leukocyte percentages in humans. Brain, behavior, and immunity 4: 339–351. 10.1016/0889-1591(90)90037-q 2092868

[43] Arntz A, Lousberg R (1990) The effects of underestimated pain and their relationship to habituation. Behaviour research and therapy 28: 15–28. 2302146 10.1016/0005-7967(90)90051-j

[44] Oka S, Chapman CR, Kim B, Shimizu O, Noma N, et al (2010) Predictability of painful stimulation modulates subjective and physiological responses. The journal of pain: official journal of the American Pain Society 11: 239–246. 19853519 10.1016/j.jpain.2009.07.009

[45] Weiss JM (1968) Effects of coping responses on stress. Journal of comparative and physiological psychology 65: 251–260. 5668311 10.1037/h0025562

[46] Maier S, Watkins L (1998) Stressor Controllability, Anxiety, and Serotonin. Cognitive Therapy and Research 22: 595–613.

[47] Koolhaas JM, Bartolomucci A, Buwalda B, de Boer SF, Flugge G, et al (2011) Stress revisited: a critical evaluation of the stress concept. Neuroscience and biobehavioral reviews 35: 1291–1301. 21316391 10.1016/j.neubiorev.2011.02.003

[48] Arntz A, van Eck M, de Jong P (1991) Avoidance of pain of unpredictable intensity. Behaviour research and therapy 29: 197–201. 2021382 10.1016/0005-7967(91)90048-8

[49] Myers ND, Brincks AM, Ames AJ, Prado GJ, Penedo FJ, et al (2012) Multilevel modeling in psychosomatic medicine research. Psychosomatic medicine 74: 925–936. 23107843 10.1097/PSY.0b013e3182736971 PMC3498540

[50] Vossen H, Van Breukelen G, Hermens H, Van Os J, Lousberg R (2011) More potential in statistical analyses of event-related potentials: a mixed regression approach. International journal of methods in psychiatric research 20: e56–68. 21812066 10.1002/mpr.348 PMC6878471

[51] Bromm B, Meier W (1984) The intracutaneous stimulus: a new pain model for algesimetric studies. Methods and findings in experimental and clinical pharmacology 6: 405–410. 6503475

[52] Karsdorp PA, Ranson S, Schrooten MG, Vlaeyen JW (2012) Pain catastrophizing, threat, and the informational value of mood: task persistence during a painful finger pressing task. Pain 153: 1410–1417. 22542915 10.1016/j.pain.2012.02.026

[53] Endler NS, Kocovski NL (2001) State and trait anxiety revisited. Journal of anxiety disorders 15: 231–245. 11442141 10.1016/s0887-6185(01)00060-3

[54] Traue HC, Gottwald A, Henderson PR, Bakal DA (1985) Nonverbal expressiveness and EMG activity in tension headache sufferers and controls. Journal of psychosomatic research 29: 375–381. 4057125 10.1016/0022-3999(85)90023-6

